# Dataset of breakthrough time for various modified sand materials using Rhodamine-B as an adsorbate

**DOI:** 10.1016/j.dib.2019.104751

**Published:** 2019-11-12

**Authors:** Pratik Kumar, Satinder Kaur Brar, Maximiliano Cledon, Azadeh Kermanshahi-pour, Rosa Galvez-Cloutier

**Affiliations:** a INRS-ETE, Université du Québec, 490, Rue de la Couronne, Québec, G1K 9A9, Canada; b Department of Civil Engineering, Lassonde School of Engineering, York University, North York, Toronto, M3J 1P3, Ontario, Canada; c CIMAS (CONICET, UnComa, Rio Negro), Güemes 1030, San Antonio Oeste, Rio Negro, Argentina; d Biorefining and Remediation Laboratory, Department of Process Engineering and Applied Science, Dalhousie University, 1360 Barrington Street, Halifax, Nova Scotia, B3J 1Z1, Canada; e Faculté des Sciences et Génie, Département de génie Civil et génie des Eaux, Université Laval, Québec (Québec), Canada

**Keywords:** Rhodamine-B, Sand media, Graphitized sand, Manganese-coated sand, Adsorption, Breakthrough time

## Abstract

Removal of synthetic dyes from wastewater generated by the textile industries is important. Rhodamine-B is widely used colorant and is medically proven to lead to tissue borne sarcoma, reproductive and neurotoxicity issues in humans, if still present in the treated drinking water. Herein, this dataset provides information on different forms of sand materials for their effective utilization as an adsorbent material for Rhodamine-B. The effectiveness of the media was measured in terms of breakthrough time obtained. One of the 27 presented data set is a part of a research article [1] explaining the breakthrough time of these filter media under specific experimental condition. All these data is a combination of three variables that were studied: a) concentration of Rhodamine-B (1 mg/L, 5 mg/L and 10 mg/L), b) flow velocity of Rhodamine-B spiked water (2 mL/min, 5 mL/min and 10 mL/min) and c) bed height (7.5 cm, 10 cm, and 12.5 cm). At any bed height, the breakthrough time of graphitized sand (brewery sugar coated, GS1) was found to be 3–4 times higher than the second best adsorbent, i.e., manganese dioxide coated on GS1.

**Table:** Specifications Table

Subject area	Chemical Engineering
More specific subject area	Filtration and separation
Type of data	Tables
How data was acquired	Spectrophotometer reading of 96-well plates at a wavelength of 550 nm. A calibration graph was plotted based on the standard (known) concentration of Rhodamine-B (adsorbate) and the optical density to determine the filtered Rhodamine-B concentration. The breakthrough time (effluent concentration becomes 5% of initial adsorbate concentration) was noted.
Data format	Raw data as obtained from the experimental observation and predicted value from bed depth service time model equation
Experimental factors	Variables: a) concentration of adsorbate, b) flow velocity of influent to the filter containing adsorbent and c) bed height of adsorbent in the filter column Adsorbents: Raw sand (RS), Graphitized sand using brewery effluent as the sugar source (GS1), Graphitized sand using sucrose as the sugar source (GS2), manganese dioxide impregnated raw sand (RSMN) and graphitized sand (GS1M, GS2M)
Experimental features	Data of breakthrough time using different sand forms such as raw sand, graphitized sand, manganese-coated sand. A laboratory-scale model filter column was used for the filtration experiments.
Data source location	INRS-ETE, Université du Québec, 490, Rue de la Couronne, Québec, Canada G1K 9A9
Data accessibility	Data presented in these articles
Related research article	Kumar, P., Rehab, H., Hegde, K., Brar, S. K., Cledon, M., Kermanshahi-pour, A., . . . Surampalli, R. Y. (2020). Physical and biological removal of Microcystin-LR and other water contaminants in a biofilter using Manganese Dioxide coated sand and Graphene sand composites. Science of The Total Environment, 703. doi: https://doi.org/10.1016/j.scitotenv.2019.135052 [1].

**Table:** 

**Value of the Data** • The dataset presented in this article summarizes the breakthrough time of six different filter media used as an adsorbent material. The data set will help researchers to get insight into the different filter media that can prove as an alternative to the raw sand media (conventionally used). • A more in-depth comparison can be made as to the data deals with three prominent variables (flow rate, adsorbate concentration, and adsorbent bed height) that are expected for any filter adsorbent studies. These variables will help the water treatment scientists to explore more possibilities for its utility. • Overall, this dataset can expedite the scientific community in gathering more insights into various other filter adsorbents apart from conventional sand media that proved to be more effective in adsorbing Rhodamine-B (adsorbate). • These data in the form of breakthrough time can set a benchmark for adsorption study of other micropollutants as well where reference to this dataset can be made in the future to support the technical explanation. • Any scale-up filter column study using these filter media can be extrapolated using these data as they closely fit with the bed-depth service time model (BDST). This model is widely applied to predict the breakthrough time under different experimental conditions in a filter column study.

## 1 Data

The dataset comprises experimental data that were obtained for all the six filter adsorbents in terms of their breakthrough time period at the specified concentration of the adsorbate (3-levels, more detail in section 2), bed height of the adsorbent (3-levels, more detail in section 2) and flow through velocity (3-levels, more detail in section 2). Table 1 tabulates breakthrough time data for an initial adsorbate (Rhodamine-B) concentration of 1 mg/L for all six adsorbents. Similarly, Table 2 and Table 3 tabulates the breakthrough time period for the initial adsorbate (Rhodamine-B) concentration of 5 mg/L and 10 mg/L, respectively for all six adsorbents.

**Table 1:** Breakthrough time data for all the adsorbent material at the initial adsorbate (Rhodamine-B) concentration of 1 mg/L.

Bed Depth	Conditions	RS	RSMN	GS1	GS2	GS1MN	GS2MN
Z = 7.5 cms	Uo (cm/min)	1.56	1.56	1.56	1.56	1.56	1.56
	Tb (min)	3.67	4.08	51	4.67	21	5
	Uo (cm/min)	0.78	0.78	0.78	0.78	0.78	0.78
	Tb (min)	59	80	5007	377	1678	431
	Uo (cm/min)	0.31	0.31	0.31	0.31	0.31	0.31
	Tb (min)	227	310	8040	1508	6702	1721
Z = 10 cms	Uo (cm/min)	1.56	1.56	1.56	1.56	1.56	1.56
	Tb (min)	22	29	1702	129	573	147
	Uo (cm/min)	0.78	0.78	0.78	0.78	0.78	0.78
	Tb (min)	96	131	8312	626	2782	714
	Uo (cm/min)	0.31	0.31	0.31	0.31	0.31	0.31
	Tb (min)	320	438	10355	2133	9481	2435
Z = 12.5 cms	Uo (cm/min)	1.56	1.56	1.56	1.56	1.56	1.56
	Tb predicted (min)	41	55	3355	253	1126	289
	Tb observed (min)	36	59	3165	238	1078	265
	Uo (cm/min)	0.78	0.78	0.78	0.78	0.78	0.78
	Tb predicted (min)	133	181	11617	874	3887	998
	Tb observed (min)	125	195	10584	901	3387	967
	Uo (cm/min)	0.31	0.31	0.31	0.31	0.31	0.31
	Tb predicted (min)	413	565	36671	2758	12260	3149

Tb= Breakthrough Time; Uo = Linear flow through velocity; Z = Bed depth; RS: Raw sand; RSMN: Raw sand manganese; GS1: Brewery solution sugar coated sand; GS2: Sucrose solution coated sand; GS1M and GS2M: Manganese dioxide-coated graphitized sand from respective sugar sources.

**Table 2:** Breakthrough time data for all the adsorbent material at the initial adsorbate (Rhodamine-B) concentration of 5 mg/L.

Bed Depth	Conditions	RS	RSMN	GS1	GS2	GS1MN	GS2MN
Z = 7.5 cms	Uo (cm/min)	1.56	1.56	1.56	1.56	1.56	1.56
	Tb (min)	0.74	0.82	10	0.94	4.2	1
	Uo (cm/min)	0.78	0.78	0.78	0.78	0.78	0.78
	Tb (min)	12	16	1001	75	336	86
	Uo (cm/min)	0.31	0.31	0.31	0.31	0.31	0.31
	Tb (min)	45	62	4008	302	1340	344
Z = 10 cms	Uo (cm/min)	1.56	1.56	1.56	1.56	1.56	1.56
	Tb (min)	4	6	340	26	115	29
	Uo (cm/min)	0.78	0.78	0.78	0.78	0.78	0.78
	Tb (min)	19	26	1662	125	556	143
	Uo (cm/min)	0.31	0.31	0.31	0.31	0.31	0.31
	Tb (min)	64	88	5671	427	1896	487
Z = 12.5 cms	Uo (cm/min)	1.56	1.56	1.56	1.56	1.56	1.56
	Tb predicted (min)	8	11	671	51	225	58
	Tb observed (min)	9	13	621	44	211	64
	Uo (cm/min)	0.78	0.78	0.78	0.78	0.78	0.78
	Tb predicted (min)	27	36	2323	175	777	200
	Tb observed (min)	24	39	2231	171	719	182
	Uo (cm/min)	0.31	0.31	0.31	0.31	0.31	0.31
	Tb predicted (min)	83	113	7334	552	2452	630

Tb= Breakthrough Time; Uo = Linear flow through velocity; Z = Bed depth; RS: Raw sand; RSMN: Raw sand manganese; GS1: Brewery solution sugar coated sand; GS2: Sucrose solution coated sand; GS1M and GS2M: Manganese dioxide-coated graphitized sand from respective sugar sources.

**Table 3:** Breakthrough time data for all the adsorbent material at the initial adsorbate (Rhodamine-B) concentration of 10 mg/L.

Bed Depth	Conditions	RS	RSMN	GS1	GS2	GS1MN	GS2MN
Z = 7.5 cms	Uo (cm/min)	1.56	1.56	1.56	1.56	1.56	1.56
	Tb (min)	0.37	0.41	5	0.47	2.1	0.5
	Uo (cm/min)	0.78	0.78	0.78	0.78	0.78	0.78
	Tb (min)	6	8	501	38	168	43
	Uo (cm/min)	0.31	0.31	0.31	0.31	0.31	0.31
	Tb (min)	23	31	2004	151	670	172
Z = 10 cms	Uo (cm/min)	1.56	1.56	1.56	1.56	1.56	1.56
	Tb (min)	2	3	170	13	57	15
	Uo (cm/min)	0.78	0.78	0.78	0.78	0.78	0.78
	Tb (min)	10	13	831	63	278	71
	Uo (cm/min)	0.31	0.31	0.31	0.31	0.31	0.31
	Tb (min)	32	44	2836	213	948	244
Z = 12.5 cms	Uo (cm/min)	1.56	1.56	1.56	1.56	1.56	1.56
	Tb predicted (min)	4	5	335	25	113	29
	Tb observed (min)	4	5	311	22	101	32
	Uo (cm/min)	0.78	0.78	0.78	0.78	0.78	0.78
	Tb predicted (min)	13	18	1162	87	389	100
	Tb observed (min)	14	21	1098	92	356	99
	Uo (cm/min)	0.31	0.31	0.31	0.31	0.31	0.31
	Tb predicted (min)	41	56	3667	276	1226	315

Tb= Breakthrough Time; Uo = Linear flow through velocity; Z = Bed depth; RS: Raw sand; RSMN: Raw sand manganese; GS1: Brewery solution sugar coated sand; GS2: Sucrose solution coated sand; GS1M and GS2M: Manganese dioxide-coated graphitized sand from respective sugar sources.

## 2 Experimental design, materials, and methods

### 2.1 Preparation of adsorbent media used

The preparation method for obtaining graphitized-sand (GS1 and GS2) and manganese-coated sand (RSMN, GS1M, and GS2M) is described by Gupta et al., 2012 [2] and Jia et al. (2015) [3], respectively. Raw sand (quartz sand) was obtained from Chemin Ste-Foy DWTP, Quebec City, Canada. The sugar solution (brewery effluent for GS1 and sucrose for GS2) was prepared according to strength 0.1g/g-sand and was caromalized at 186 °C followed by graphitization at 600 °C for 3 hours under reduced atmospheric condition. To obtain GS1M and GS2M, potassium permanganate (7%, w/v) was melted (240 °C) over the graphitized sand and kept for 3 hours before washing and drying them at room temperature overnight.

### 2.2 Breakthrough time study

Rhodamine-B was prepared in three different concentration viz. 1 mg/L, 5 mg/L, and 10 mg/L. Other two variables were bed height of the adsorbents: 7.5 cm, 10 cm, 12.5 cm, and linear flow through velocity: 2 mL/min, 5 mL/min and 10 mL/min. Hence, a total of 27 different experimental combinations were obtained for each of the six adsorbents. The filter column used for the adsorption experiment has an internal diameter of 2.3 cm and a total height of 20 cm. After the passage of every 40 mL (∼twice the bed volume) at a specific flow rate and bed height, the optical density (OD) of the collected filtered-effluent underwent spectrophotometric reading at the wavelength of 550 nm. All the tests were repeated 3 times. Based on the standardized relationship obtained between OD and initial Rhodamine-B (adsorbate) concentration (Co), the effluent concentration (Ct) was determined. At Ct/Co ratio of 0.05, the time was noted and is referred to as the ‘breakthrough time period’.

It was observed that for the specific initial Rhodamine-B concentration and flow rate, the relationship between the bed height and breakthrough time followed the bed-depth service time model (BDST model). The model is represented in the form of Equation (1) as under:

(1) $$T_{b}=\frac{N_{0}Z}{UoCo}-\frac{1}{k_{bd}C_{0}}\ln\left(\frac{C_{0}}{C_{B}}-1\right)$$

where No is the saturation concentration per unit bed volume (mg/L), Z is the bed height, Co is influent concentration, Uo is flow through velocity, k_bd_ is the adsorption rate constant (L/mg.min) and C_B_ is the concentration of adsorbate at the breakpoint.

It can be observed from Table 1, Table 2, Table 3 that as the bed depth increases, there is an increase in breakthrough time (Tb) as well, while Tb decreases as the initial concentration of Rhodamine-B increases. Overall the trend of breakthrough time followed the order: RS < RSMN < GS2 < GS2M < GS1M < GS1 where GS1 showed almost three times more Tb than second best adsorbent, i.e., GS1M (for any similar chosen experimental condition). All the filter media performed better than the raw sand attributing an increase in roughness, active adsorption sites and surface area thereby assisting more Rhodamine-B molecule adsorption [4]. Table 1, Table 2, Table 3 also shows the predicted breakthrough time using BDST model at highest bed depth value of 12.5 cm under all three flow rates (10 mL/min, 5 mL/min and 2 mL/min) and under every different initial Rhodamine-B concentration (1 mg/L, 5 gm/L and 10 mg/L). These predicted values were compared with the observed experimental values to cross-check the linearity of the BDST model. The experimental and the predicted values are mentioned in Table 1, Table 2, Table 3. The kinetics data of these adsorbents in relation to the metal adsorption has been mentioned in Kumar et al. (2020) [1].
